# Advances in Matrix-Supported Palladium Nanocatalysts for Water Treatment

**DOI:** 10.3390/nano12203593

**Published:** 2022-10-13

**Authors:** Wenhu Wang, Mallikarjuna N. Nadagouda, Sharmila M. Mukhopadhyay

**Affiliations:** 1 Frontier Institute for Research in Sensor Technologies (FIRST), The University of Maine, Orono, ME 04469, USA; 2 Graduate School, Wright State University, Dayton, OH 45435, USA; 3 Department of Mechanical Engineering, The University of Maine, Orono, ME 04469, USA

**Keywords:** palladium nanocatalyst, hybrid catalyst-support systems, water purification

## Abstract

Advanced catalysts are crucial for a wide range of chemical, pharmaceutical, energy, and environmental applications. They can reduce energy barriers and increase reaction rates for desirable transformations, making many critical large-scale processes feasible, eco-friendly, energy-efficient, and affordable. Advances in nanotechnology have ushered in a new era for heterogeneous catalysis. Nanoscale catalytic materials are known to surpass their conventional macro-sized counterparts in performance and precision, owing it to their ultra-high surface activities and unique size-dependent quantum properties. In water treatment, nanocatalysts can offer significant promise for novel and ecofriendly pollutant degradation technologies that can be tailored for customer-specific needs. In particular, nano-palladium catalysts have shown promise in degrading larger molecules, making them attractive for mitigating emerging contaminants. However, the applicability of nanomaterials, including nanocatalysts, in practical deployable and ecofriendly devices, is severely limited due to their easy proliferation into the service environment, which raises concerns of toxicity, material retrieval, reusability, and related cost and safety issues. To overcome this limitation, matrix-supported hybrid nanostructures, where nanocatalysts are integrated with other solids for stability and durability, can be employed. The interaction between the support and nanocatalysts becomes important in these materials and needs to be well investigated to better understand their physical, chemical, and catalytic behavior. This review paper presents an overview of recent studies on matrix-supported Pd-nanocatalysts and highlights some of the novel emerging concepts. The focus is on suitable approaches to integrate nanocatalysts in water treatment applications to mitigate emerging contaminants including halogenated molecules. The state-of-the-art supports for palladium nanocatalysts that can be deployed in water treatment systems are reviewed. In addition, research opportunities are emphasized to design robust, reusable, and ecofriendly nanocatalyst architecture.

## 1. Introduction and Background

Chemical and biological contaminants in water pose a significant threat to public health. Emerging contaminants including pesticides, textile dyes, plasticizers, disinfection by-products, polychlorinated biphenyls (PCBs), polycyclic aromatic hydrocarbons (PAHs), and fluorinated compounds such as perfluorooctanoic acid (PFOA) and perfluorooctane sulfonate (PFOS), endocrine-disrupting materials, and pharmaceutical and personal care products [1,2,3,4,5,6] are relatively stable and do not naturally break down in the environment. Conventional water treatment technologies such as adsorption, ion exchange, and membrane filtration [7,8,9,10,11] have shown to be ineffective in removing these contaminants. In addition, these processes generate waste or sludge streams that need to be further treated and disposed. The spent adsorbent, resins, and membrane concentrate need to be either regenerated or incinerated, to avoid secondary contamination. In some cases, destructive options break the C−C bonds in the molecules, resulting in shorter-chain halogenated carbons that may be equally or more toxic. 

Catalytic degradation has been found to be effective in degrading halogenated molecules [12,13,14,15], which has resulted in significant research efforts devoted to developing novel nanocatalysts. Adsorption and catalytic activity of any solid is driven by its surface chemical and electrical states. In this regard, nanoscale materials provide two unprecedented advantages: (i) extremely high surface to volume ratio, which can help with high reaction rates and sensitivity in compact space; and (ii) unique surface states for tailorable optical, electrical and catalytic properties in some cases, which can lead to unprecedented catalytic behavior, leading to new reaction pathways for degradation or detection. 

Various transition and rare earth metals such as Platinum (Pt), Gold (Au), Palladium (Pd), and Rhodium (Rh) have been extensively studied as catalysts due to their high electron counts and catalytic activity. Among these catalysts, palladium, which is often regarded as an inexpensive substitute to platinum (Pt) [16], offers several advantages. Pd has high catalytic activity, and the unique ability to absorb hydrogen gas while being impervious to other gases. Particularly for water treatment applications, palladium is a promising catalyst due to this unique interaction with hydrogen. It can mitigate several emerging pollutants through reductive dehalogenation and hydrogenation reactions [17], including the persistent and challenging contaminants such as halogenated compounds. Palladium catalysts are known to convert most of these compounds into completely de-halogenated molecules. When the catalyst size is reduced to nanoscale, palladium is even more active. Palladium nanoparticles (PdNPs) have been successful in a wide range of catalytic applications including hydrogenation [18], alcohol oxidation [19], carbon–carbon bond formation [20], and electrochemical reactions in fuel cells [21]. Apart from these, Pd NPs are also employed in hydrogen storage and sensing applications [22,23]. Pd NPs can be synthesized by a variety of chemical [24], electrochemical [25], sonochemical [26], and biological [27] methods.

However, the high surface-to-volume ratio of nanocatalysts poses significant challenges during production, transportation, storage, and use. First, nanocatalysts can easily agglomerate, losing their nanoscale properties, so a large body of literature has focused on developing approaches to prevent agglomeration. Second, they can be easily dispersed into their surroundings, leading to adverse environmental and public health risks, in addition to material loss [28]. Therefore, despite their multiple advantages, large scale deployment of pure nanocatalysts may not be safe, environmentally friendly or sustainable. 

This has led to an entire field of study related to the development of hybrid materials, where the desired nanocatalyst is supported on the surface of another solid, which would prevent agglomeration and loss into the environment [29,30]. It must be underscored that physical and chemical properties of these hybrid structures relevant to catalytic activity can depend strongly on catalyst-support interactions. The basic function of all supports is to disperse and stabilize the catalytically active nanoparticles, and it is expected that the nucleation and growth mechanisms that will determine the size, shape, morphology and crystallographic facets of the final nanoparticle are determined by the nature of the substrate. Additionally, for palladium nanoparticles, it is expected that charge transfer between the noble metal and the support will significantly influence the electronic structure of the nanoparticle and therefore impact the catalytic activity of the support-catalyst complex. This has made the nature and extent of metal-support interaction a rich field by itself, for both computational and experimental studies, and the support is often used as a tool for tuning the catalyst [31,32]. Therefore, the catalyst-support hybrid material needs to be investigated holistically to understand its properties and evaluate its applicability on a case-by-case basis. 

This paper provides an overview of recent advances made in supporting palladium nanocatalysts on organic and inorganic substrates for promising catalytic applications. Special emphasis is given to palladium nanocatalyst systems for water treatment applications, especially for the degradation of emerging contaminants. These nano-hybrid materials are designed with the goal of synergizing the advantages of palladium nanocatalyst with those of the supporting solids for providing improved solutions for water sustainability.

## 2. Novel Supports for Palladium Nanoparticles and Demonstrated Applications

**Figure 1:**
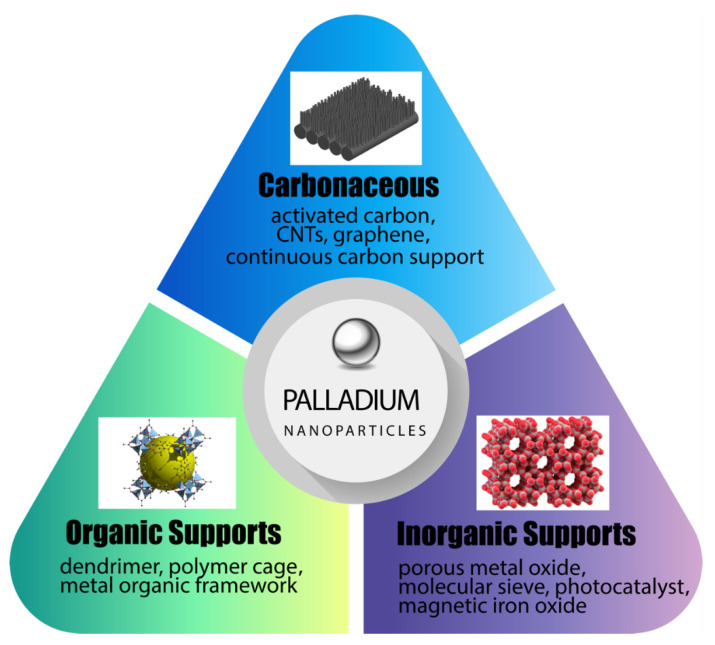
Schematic illustration of different types of support for palladium nano-catalyst.

In recent years, a variety of hybrid materials and structures have been reported where palladium nanoparticles are supported on other solid matrices including organic/polymeric, and inorganic supports [33,34]. Among the organic materials, novel supports such as dendrimers, polymer cages and metal organic framework (MOF) have been highlighted along with large porous polymers suitable for water treatment. Among inorganic supports, the focus has been on high porosity oxide systems, photocatalysts and magnetic compounds, due to their operational advantages. A third category of supports that show significant promise for supporting palladium are carbonaceous materials such as granular activated carbon (GAC), graphene and carbon nanotubes (CNT). Figure 1 provides an overview of the different substrates that can serve as a support. 

### 2.1. Novel Organic and Polymeric Supports for Pd 

Polymeric solids offer high level of flexibility and customization in shape, size, porosity (diameter of pores as well as volume fraction), and composition. Due to the wide selection of monomers available, different combinations of monomer blocks can be combined with the Pd nanoparticles, hence offering endless possibilities. Both organic and polymeric supports have shown promise in several applications related to energy [35], food industry [36], medicine [37], environment [38], and chemical catalysis [39] industries. This section will provide an overview of novel organic and polymeric supports that can be used in water-treatment applications.

#### 2.1.1. Dendrimers

Dendrimers are a family of hyper-branched polymers that are nearly spherical, sterically crowded on the exterior, and hollow in the interior [40]. The interior holes can be functionalized by organic groups such as tertiary amines, enabling them to attach very targeted metallic ions (e.g., Cu^2+^, Pd^2+^, Ni^2+^ and Pt^2+^) for catalytic or other functions. More importantly, the number of metal ions per dendrimer is nearly the same, resulting in monodispersed metal nanoparticles with finely tuned ultra-small sizes, as seen in polyamidoamine (PAMAM) -based complexes [41]. In addition, dendrimers have shown promise in “Click” chemistry, where small biocompatible molecular groups are used as lego-like building blocks to create larger structures [42]. In fact, water-soluble dendrimer templates have been created with a wide variety of ultra-small metal nanoparticles ranging from monometallic (e.g., Pt, Rh, Copper (Cu), Au, Pd, Ruthenium (Ru), and Nickel (Ni)), to bimetallic complexes (e.g., Platinum-Cobalt (Pt−Co)). These were found to be active in a series of catalytic reactions, such as Suzuki−Miyaura coupling, hydrogenation, and ammonia borane hydrolysis [43,44,45]. It was further pointed out that by varying the peripheral groups of the dendrimer, the size and shape of the metal nanoparticles can be tuned. In one study, amine functional groups resulted in more evenly distributed/shaped NPs, whereas carboxylate terminated dendrimers resulted in anisotropic NPs size distributions [46]. Eghbali et al. synthesized dendrimer-encapsulated metallic PdNPs as catalysts for hydrogen production. In this study, authors used a microwave assisted amidation method to prepare the PAMAM, followed by mixing the PAMAM dendrimers with Pd precursor (in this case, palladium acetylacetonate), finally, ammonia borane was used as the reducing agent to obtain metallic PdNPs as well as hydrogen source for methanolysis of ammonia borane. The turnover frequency (TOF) is comparable to other catalysts such as Ru or Rh. The valuable part of this study was that the authors established the relationship between PdNP particle size, dimension and dendrimer properties (e.g., dendrimer generation, core type, terminal group etc.), which could help with the design of a catalyst based on Pd and a dendrimer with higher catalytic activity. In short, it was also found that higher generation dendrimers, with a Jeffamine core and amine terminal group could yield finer PdNPs distribution and smaller particle sizes [47,48]. It is therefore expected that, with careful selection of dendrimer polymers and successful attachment of functional monomers and metal nanocatalysts, it will be possible to obtain novel water treatment functions in the near future, especially for closed systems. For example, dendrimers that can capture/release ions based on pH values of water could be used to target specific toxic metal ions. 

#### 2.1.2. Polymer Cage

**Figure 2:**
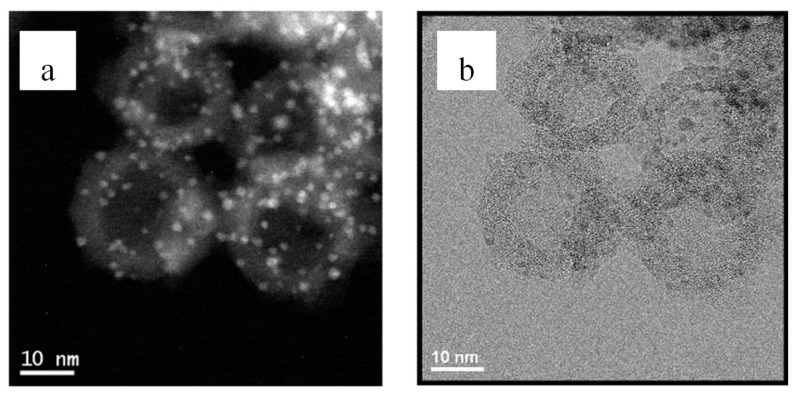
Transmission electron microscope (TEM) images of Pd cluster on silica core−shell nanospheres after removal of polymer particles: (**a**) Z-contrast; and (**b**) bright-field. “Reproduced with permission from [50], American Chemical Society, 2014”.

Polymeric cages or porous organic polymers (POPs) constitute another group of porous organic supports that have a molecular open framework structure with intrinsic inner hollows [49]. POPs offer several advantages including high surface area, structural tunability, and the option to add selected entities in the inner hollow. The inner cavities of polymer cages are generally smaller than 2 nm, which makes them suitable for encapsulating ultra-small metal nanoparticle clusters [50]. Figure 2 shows Palladium metal clusters on cage-like silica core structures formed by creating polymer particles on silica core. The polymer sites will adsorb and fix targeting metal ions onto themselves, followed by reduction in the metal ions and removal of the polymers through heat treatments in controlled atmospheres. In another study, Yang et al. [51] reported a highly active and stable Pd-based catalyst, on a reduced amine cage (RCC3), developed using a reverse double-solvent strategy. The size of the PdNPs could be controlled at precisely 0.72 nm and further characterization tests revealed that the PdNPs are in a metallic state, with over 70% of them located inside the cage. This synthesized catalyst showed very high catalytic activity and stability in hydrogen generation from ammonia borane, formation of 4-aminophenol from hydrogenation of nitroarenes, and hydrogen reduction in Congo red dyes. Tao et al. [52] also explored the possibility size tuning the PdNPs encapsulated polymer cage (Phos-cage) from 0.8 nm to 3.3 nm by changing the organic solvent. The size dependence of PdNPs in the reduction in p-nitrophenol was investigated, and the authors found that the catalytic activity increases as the PdNPs size decreases. All Pd-complex variants with different PdNPs sizes showed over 95% activity after 5 cycles of durability tests. 

A major advantage of this Pd/POP catalyst is the open structure, which can boost the interaction of the nanocatalyst PdNPs with the reactant [53]. Additionally, by encapsulating the metallic cluster in the polymer cages that have suitable properties, the heterogeneous catalysts may be dispersed in a solution to behave like a homogeneous catalyst, which may improve their kinetic performance in the desired catalytic application. Recent studies indicate that polymer cage-related liquid-phase catalysis may be utilized in selected water-treatment reactors. Sun et al. showed designable POP structures for improved selectivity and capacity of target species. The functionalized organic backbone could be designed to interact with target analyte via a different adsorption mechanism (e.g., ion-exchange, coordinative complexion, host-guest interaction) by using the De novo synthesis. Moreover, a well-tuned POP structure could allow the contaminant to enter and travel inside the pores, and interact with the functional groups at higher level [54]. However, it must be underscored that such an approach will only be practical and sustainable if the cages can be retrieved from water after use.

#### 2.1.3. Porous Metal-Organic Frameworks (MOFs) 

Metal-organic frameworks (MOFs) have attracted significant attention in recent years due to their molecular adsorption and chemical separation capacity and chemical sensing potential. MOFs have shown to be effective for anchoring PdNPs due to their large surface area, tunable structure, porosity, and their rapid electron transport pathways that can improve the catalytic activities of PdNPs [55,56,57]. Ri et al. [58] synthesized manganese-cerium supported Pd catalyst by calcining Mn/Ce-MOF at 300 °C first in Ar and then in O_2_ for 2 h each. The resulting catalyst was successful in completely oxidizing toluene at 190 °C with good water resistance (up to 10 vol%) and reusability. In another study, Lin et al. synthesized a Pd/Ce MOF system through microwave irradiation, resulting in a Pd/Ce –MOF catalyst structure. This material showed a unique property of oxidizing CO at 92 °C and CO_2_ capture at 0 °C [59]. 

Pd/MOFs have also shown potential to improve reaction efficiency and activity in cross-coupling reactions, where palladium is the key catalyst. Palladium catalyst is particularly important for cross-coupling reaction, where it displays high selectivity, yields and reaction speed, as well as high stability towards moisture and air [60,61,62]. Carson et al. synthesized a Pd encapsulated chromium terephthalate MOF, MIL-101-NH_2_ (Cr) (MIL, Matérial Institut Lavoisier) catalyst with well dispersed PdNPs (average size = 1 nm). This material facilitated Suzuki–Miyaura cross-coupling reaction of aryl bromides with boronic acids to a higher yield in a shortened time compared to other catalysts. Moreover, a durability test was also carried out to examine the robustness of the catalyst. It was pointed out that the fluoride base maintained the MOF structure to avoid leaching. [63] 

### 2.2. Advances in Inorganic and Ceramic Supports for Palladium Nanocatalysts

Inorganic supports (e.g., ceramic oxides and nitrides) have shown promise in enhancing palladium nanocatalysts due to their higher thermal and chemical stabilities compared to polymeric supports. Additionally, the chemical and electronic properties of many metal oxides can be easily tailored by adding dopants or functionalizing their surfaces, providing options for tuning the catalyst-support interactions [64,65,66,67,68]. Three types of supports that show future promise in water treatment applications are highlighted in the following section: (1) inert high porosity supports (e.g., zeolites and silica); (2) surface-active photocatalytic supports (e.g., titania and nitrides); and (3) magnetic oxides that offer enhanced capabilities for separation after use [66,67,68].

#### 2.2.1. Porous Oxide for Supporting Palladium 

Several high porosity oxide materials including zeolites (i.e., crystalline microporous alumino-silicate minerals) and mesoporous silica materials (i.e., amorphous silica with a regular arrangement of mesopores) [69] have been used for catalysis, gas storage and separation, energy storage, light emission, and other applications, including use in aqueous systems like aquaria [70]. By stabilizing PdNPs onto a zeolite matrix, high reaction activity for methane oxidation was shown in treatment of exhaust fumes [71,72]. In this study, common challenges in methane oxidation are catalyst sintering and deactivation. These issues were solved by post-exchange with sodium and confining the PdNPs inside the zeolite, as the catalyst is able to maintain good performance without deactivation for over 90 hrs exposure to the steam [72].

Additionally, highly active and stable PdNPs on zeolite/silica molecular sieves have shown remarkable selectivity in hydrogenation [73,74]. Li et al. reported Pd supported on molecular sieves for hydrogenation reactions. They could evenly disperse the Pd catalyst at the atomic level, and these were very effective for converting various aromatic alkynes into their corresponding alkenes. More importantly, the authors could scale up this synthesis. Ten centimeter diameter discs were prepared, which did not show any NP agglomeration. The conversion loss is controlled at 5%, and the sample catalytic activity remained the same even after a year, which makes it a potential candidate for larger industrial applications [75]. Palladium/zeolite catalysts have also been extensively used in C−C cross-coupling reactions [76]. Wang et al. reported a novel release/capture mechanism for Pd on the ultra-stable Y (USY) zeolite-supported Pd catalysts for C−C cross-coupling reaction. This was proposed as a highly dynamic catalyst that can release the Pd into the surrounding environment and then successfully recover them back on the zeolite surface. The idea is to provide homogeneous catalysis, which can potentially provide higher reaction rate. The authors claimed the palladium will exist in the form of Pd^2+^ ions, which are too large to escape far from the surface. In the presence of a strong base, they can be easily re-adsorbed by the anionic framework of the zeolite. This latter step is achieved by adding tetrapropylammonium hydroxide as the base after the coupling reaction is over. A reusability test was also carried out and minimum Pd content loss was observed after being used ten times [77].

These porous oxide-Pd complexes can be very useful for water treatment applications. Again, these are also particulate materials, which need to be packaged into carefully designed columns so that they do not wash out with the water during use. 

#### 2.2.2. Photocatalyst-Supported Palladium

A significant body of research has been conducted to show that Pd-enhanced photoactive materials can harness light energy for select chemical reactions [78,79,80,81]. Verma et al. stated PdNPs could be dispersed onto titanium cluster, which is a proven photocatalyst for a variety of reactions. With the addition of Pd nanodot, the bandgap of the catalyst is reduced and facilitates its activity for alcohol oxidation under visible light. This Pd-enhanced photo-catalyst shows a high yield in aerial oxidation of alcohols and high stability up to six cycles [82]. 

Nitride photocatalysts have also been used to support Pd and have benefited from that addition. Gao et al. [83] performed density function theory (DFT) calculations of single atom Pd and Pt, which are supported on graphitic carbon nitride (g-C_3_N_4_) for photon-induced CO_2_ reduction. It was predicted that these materials would reduce CO_2_ to CH_4_. Mondelli et al. performed experimental investigation using this material. They demonstrated the conversion of CO_2_ into formic acid with a base-free method, and the synthesized PdNP-based catalyst achieved a 20-fold higher activity (TOF < 95h^−1^) in comparison with other Pd/g-C3N4 catalysts, with maintained stability [84]. 

Photocatalysts are also known to be very effective in water splitting, which is a solution for fossil fuel alternatives and environmental pollution. It was shown that the introduction of PdNPs on TiO2 photocatalyst could maintain high photocatalytic activity for this reaction. Vertically aligned TiO_2_ nanotube arrays could also be functionalized with Pd nanoparticles with different particle sizes [85,86]. It has also been shown [87,88,89] that Pd nanoparticles can be uniformly distributed onto the TiO_2_ nanotube surfaces via a different method ranging from aqueous hydrothermal treatment to electrochemical anodization. For example, incipient wetness impregnation technique could deposit fine PdNPs (diameter ~10nm) onto the TiO_2_ nanotubes, besides water splitting, this synthesized heterogeneous photocatalyst could also be used for the rapid and efficient decomposition of non-biodegradable azo dyes (e.g., methyl red and methyl orange) under solar light [90].

#### 2.2.3. Magnetic Supports for Pd Nanoparticles That Can Be Retrieved after Use

As mentioned in the earlier sections, all powdery materials, either stand-alone palladium nanoparticles, or those attached to other micro-nanoscale particulates, will always present the challenge of flowing into the service environment, resulting in the loss of precious metal and potential environmental and public health risks. This has led to a large body of investigative work where nanomaterials were supported on magnetic entities, enabling magnetic separation after use [91]. 

It was shown that magnetically supported Pd can be synthesized via liquid phase dipping followed by a calcination process. Another demonstrable straightforward and scalable method is the direct deposition of PdNPs onto a magnetic support, such as Fe_3_O_4_ particles. For instance, Baig et al. successfully synthesized a magnetic PdNP based carbon-nano ferrite catalyst using a stepwise one-pot reaction. Magnetic F_3_O_4_ nanoparticles were first synthesized by mixing FeSO_4_·7H_2_O and Fe_2_(SO_4_)_3_ in water with an equal amount at pH = 10. This was followed by adding a mixture of PdCl_2_ and cellulose, and finished by a 3 h calcination treatment at 450 °C. This scalable process resulted in 20–50 nm spherical nanoparticles, which showed high catalytic activity forhydrogenation of alkenes, alkynes, and aryl nitro compounds, and could also be easily separated from the reaction products by an external magnetic field [92]. 

The other common technique for developing magnetically separable catalyst is to construct a core-shell structure, such as deposition of a layer of Pd catalysts on a magnetic core. This method prevents the aggregation issue that may occur when all components are precipitated together. Veisi et al. demonstrated a green synthesis procedure of magnetic nanocomposite with Fe_3_O_4_ as core and Pd as shell, using *Fritillaria imperialis* flower extract as a capping agent. A mixture of FeCl_2_·4H_2_O (2.0 g) and FeCl_3_·6H_2_O was used to prepare the magnetic NPs, followed by treatment with flower extract to form magnetically collectible Fe_3_O_4_@*Fritillaria* nanocomposite. Subsequent reduction in Pd^2+^ of Na_2_PdCl_4_ with Fe_3_O_4_@*Fritillaria* nanocomposite in an aqueous phase produces the final product Fe_3_O_4_@*Fritillaria/*Pd NPs. This Fe_3_O_4_@*Fritillaria/*Pd NPs could achieve a 98% reduction in nitrobenzene (4-nitrophenol) in half an hour. They were also seen to be durable and reusable for eight reaction cycles [93].

### 2.3. Carbonaceous Supports

Different types of carbon materials (especially graphitic carbon substrates with two-dimensional sp^2^ bonded carbon layers) have been used to support palladium. Graphitic carbon provides high thermal and electrical transport along the hexagonal graphite planes and also provides beneficial support for palladium catalysts. They are bio-inert and stable in both acidic and basic media, which is not true for many other supports. 

Carbonaceous supports can occur in a wide variety of structures [94,95], with the most common being carbon black, granular carbon, and activated carbon. For catalytic applications, the high surface area of the support is important for making granular activated carbons (AC) very popular as a catalyst support for the chemical industry. Carbon nanofiber (CNF) is another good candidate as a metal nanoparticle support due to their high dielectric permittivity, good flexibility, high mechanical strength and thermal stability [96,97,98]. This fibrous carbon support usually ranges from tens of nanometers to submicron in diameter. When decorated with palladium nanoparticles (e.g., PdNPs deposited on CNF), those CNF/PdNPs materials were found to be efficient and stable catalysts for a variety of applications under mild conditions (e.g., gas sensing, hydrogenation) [99,100,101]. Moreover, newly emerging nanoscale carbon materials such as carbon nanotubes (CNTs) and graphene/graphene oxide (GO) are showing very significant promise as nanocatalyst support, since they offer high specific surface area along with unique and tailorable surface electronic properties in addition to the conventional advantages of carbon. This section highlights some notable studies related to PdNP catalysts supported on activated carbon, graphene and carbon nanotubes (CNT):

#### 2.3.1. Activated Carbon

**Figure 3:**
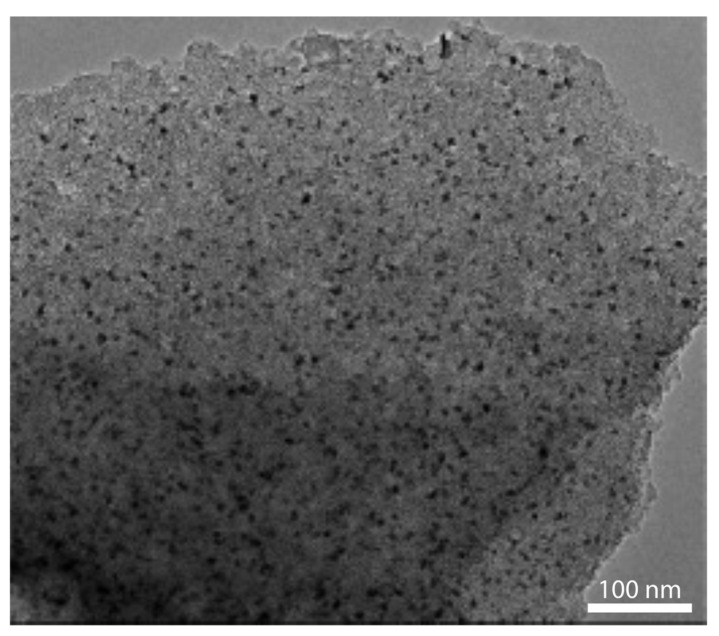
TEM images of ALD Pd nanoparticles supported on the AC undergoing HNO_3_ etching and then calcined at 800 °C. “Reproduced with permission from [103]. American Chemical Society, 2015”.

Activated carbon (AC), which can be produced from inexpensive raw materials such as coal, coconut, bamboo and other forms of vegetation via physical activation or chemical activation, has been demonstrated as a useful supporting material for Pd [102,103,104]. For instance, Gong et al. [103] used the atomic layer deposition (ALD) technique to synthesize PdNPs on an AC substrate. Uniform size distribution of the PdNPs was seen throughout the entire support. The surface chemistry of AC was tuned via heat and nitric acid treatments, and it was reported that the size and loading of PdNPs could be controlled through ALD cycles. Figure 3 shows TEM images of ALD deposited Pd-NP obtained by one cycle of Pd ALD on the AC support treated with HNO_3_ and then calcined at 800 °C. 

The effectiveness of these catalysts in sensing and detection technologies has been reported by several authors. For instance, Veerakumar et al. reported use of PdNP supported on porous AC for heavy metal detection via electrochemical method (cyclic voltammetry). With 1.5 wt% Pd loading, the Pd/AC material was seen to have high selectivity and sensitivity [104]. 

#### 2.3.2. Graphene 

Graphene, which can be defined as a single layer of graphitic carbon, has attracted attention in catalysis due to its unique two-dimensional structure, intrinsic carrier concentration, active surface area, and promising mechanical and thermal stability. Lower-cost and easier to produce derivatives of graphene are graphene oxide (GO) and reduced graphene oxide (RGO). They have stronger interactions with palladium due to higher concentration of defect sites and oxygen-based functional groups [105]. For instance, Elazab et al. [106] reported creating several varieties of graphene supported Pd nanocatalysts by microwave-assisted chemical reduction in aqueous suspension of Pd(NO_3_)_2_ and graphene sheets. This method combines strong electrostatic adsorption (SEA) with a solventless microwave reduction method of Pd precursors. Four different types of commercially available graphene supports (graphene powder 1–5 layers, GO, RGO, and graphene nanoplatelet aggregates) were used to make different grades of hybrid catalyst. 

The band structure of graphene can be tailored by doping graphene with other atoms. This can enable modulation of electronic properties, and manipulation of surface chemistry in graphene for targeted applications. For example, Movahed et al. prepared novel nitrogen-doped graphene (NG) by using urea as a nitrogen source. PdNPs were anchored to the surface of the NG by a reduction reaction of PdCl_2_ [107]. The catalytic activity of these materials was examined in a Suzuki cross-coupling reaction of several aryl boronic acids with various aryl halides. Reactions were performed with PdNPs-NG (0.025 mol% of Pd) in EtOH/H_2_O mixture (1:1 ratio) using K_2_CO_3_ as a base at 60 °C. The results showed that aryl halides, with either electron-donating or electron-withdrawing substituents, efficiently reacted with aryl boronic acids in the presence of these catalysts.

#### 2.3.3. Carbon Nanotubes

Carbon nanotubes can be regarded as cylinders of rolled-up graphene sheets and show significant promise as nano-carbon support for catalytic materials. [108,109] 

CNTs have been used as supports for PdNPs by several investigators [110,111,112,113]. For instance, Yoon et al. [114] prepared CNT-supported PdNPs by reducing Pd(II)hexafluoroacetylacetone with hydrogen in the presence of multi-walled carbon nanotubes (MWCNTs) under supercritical fluid CO_2_ at 80 °C and 150 atm. Additional surface functionalization steps (e.g., molecular attachments or coatings) were often used to improve the attachment of PdNPs on the CNTs [115,116]. Nie et al. [117] used an in-situ polymerization approach, which involved polymerization of aniline to create Polyaniline (PANI) on the surface of MWCNT at 0 to 5 °C prior to Pd deposition. PANI acted as a reducing agent in this process, while enabling the stabilization of PdNPs. 

It is clear that carbon-based nanomaterials provide significant advantages as support for Pd nanoparticles. However, as mentioned earlier, hybrid materials comprising of stand-alone nanocarbon-supported Pd have challenges related to a lack of integrity, catalyst leaching and dispersion. This challenge has motivated some investigators to incorporate nanoscale materials into larger monolithic solids. Yuan et al. developed a two-step strategy to produce a novel Pd/CNT-SiC monolith catalyst. A SiC foam was coated with a layer of CNT, with PdNPs dispersed on the surface. The coupling between iodobenzene and phenylboronic acid catalyzed by the Pd/CNT-SiC monolith provided nearly 100% conversion after 60 min of reaction at 60 °C. The turnover frequency (TOF) was reported to be 1800 h^−1^, which is approximately four times greater than that of the reaction with isolated Pd/CNTs and 22 times greater than that of the reaction with Pd/SiC foam without CNT under the same conditions. The high activity of the catalyst was attributed to the nano-structured CNT-SiC monolith that offered higher surface area [118,119]. Since this study did not further analyze the reusability of this material after repeated use, it is not possible to comment on the robustness of this hybrid material, or its applicability in water treatment systems.

## 3. Supports for Pd Nanocatalysts Developed Specifically for Water Remediation

Recent years have seen widespread contamination of water with halogenated compounds and unsaturated hydrocarbons, and catalytic degradation may be one of the most eco-friendly options of mitigating them. Palladium may be one of the most versatile catalysts for water treatment via a catalytic reductive method [120,121,122]. These catalysts can enable reductive dehalogenation and/or hydrogenation, which has several advantages compared to other water purification techniques. First, a broad range of compounds (such as halogenated alkanes and aromatics, oxidized nitrogen, etc.) can be degraded in aqueous solution at ambient temperatures by Pd catalysts. Additionally, the reaction can be rapid and complete, resulting in a reduction in the larger and more toxic compound into more benign molecules, leaving no intermediates. Lastly, reductive degradation consumes less energy and causes no secondary pollution, as seen in combustion processes. 

These potential advantages have motivated the authors of this paper to focus their efforts on matrix supported Pd nanocatalysts for water treatment systems. Based on how the catalytic material can be introduced in the water, these results have been classified as “particulate materials,” which include powders or pellets that need to be enclosed in packages or columns, and “continuous larger membranes”, which can be applied directly in the water. 

### 3.1. Particulate Supports for Pd-Based Catalysts

Particulate Pd-based catalysts for water treatment have been developed in various forms, such as surfactant-directed assemblies, magnetically supported systems, and cellulose-derived supported structures. 

Huang et al. [123] reported a one-pot surfactant-directed approach of synthesizing isolated PdNPs to maximize their catalytic activity, by using cetyl-trimethyl-ammonium bromide (CTAB) as the surfactant. The shape of synthesized PdNPs showed a clear dependence on the CTAB concentration. The resulting Pd catalyst exhibits strong catalytic activity for degradation of 4-chlorophenol (4-CP) in water. It was also shown that the catalyst could maintain 87% efficiency after eight repeated runs. However, a gradual agglomeration of PdNPs was observed after each de-chlorination cycle, which would be a problem in practical applications.

Luo et al. [124] used a one-step green synthesis of Fe-Pd NPs via plant extract, which works as both reducing and capping agents. The prepared bimetallic Fe/Pd nanoparticles were used to degrade Orange II (model azo dye) in water. The bimetallic system showed a 98% removal in 12 h while its counterpart Fe NPs without the Pd showed an only 16% removal. A batch test also showed positive dependence of catalyst loading and temperature, while negative dependence of Orange II concentration and pH values (higher pH values lower removal efficiency).

Nadagouda et al. [125] synthesized polymer-supported Pd-nanocatalysts for autocatalytic reduction in a range of emerging contaminants: methylene blue; octylphenoal; atrazine; and triclosan. The materials consisted of polypyrrole-coated cellulose fibers with PdNPs decoration and showed a high level of porosity. Different shapes of the nanocrystals (such as cubes, prisms, and spheres) could be formed based on the polypyrrole thickness. Very high reduction rates (ranging from 0.0183 to 0.1168 M^−1^s^−1^) were observed at room temperature. 

Li et al. [126] performed experimental and theoretical studies on PdNPs decorated nitrogen-doped grapheme (Pd/NG) for degradation of halogenated tetrabromobisphenol A (TBBPA), and triclosan (TCS). Pd/NG demonstrated better removal results compared to Pdon undoped Graphene (Pd/G) and Pd supported on commercial carbon (Pd/C). DFT calculations were carried out showing that the adsorption and degradation of TBBPA on Pd/NG surfaces would more energy efficient than on Pd/G; the TBBPA activation energy on Pd/NG was 2.7 eV, compared to 2.8 eV on Pd/G surface. Meanwhile, Darabdhara et al. [127] designed a bimetallic nanoparticle system of Au-Pd on reduced graphene oxide (rGO) for photocatalytic degradation of organic pollutants (phenol, 2-chlorophenol, 2-nitrophenol). Reduction efficiencies of 94.4, 97 and 98.6%, respectively, were observed. 

Even though PdNPs are known to be effective and efficient for water treatment due to their high activity and selectivity, catalyst poisoning is always a concern in water treatment processes. Comandella et al. [128] made an effort to avoid Pd catalyst poisoning by embedding Pd/Al_2_O_3_ particles in Poly(dimethylsiloxane)(PDMS). The encapsulation would normally be expected to sacrifice some activity in return of longer catalyst life compared with pure PdNPs. However, comparison of catalytic activities with and without the encapsulation has not been reported, and hence cannot be quantified. What was seen was that the PDMS-embedded Pd/Al_2_O_3_ could maintain its full initial activity for up to over 750 cycles during the degradation of trichloroethene.

### 3.2. Pd Nanocatalysts on Continuous Solid Structures Suitable for Water Treatment

As mentioned in earlier sections, isolated or particulate catalysts would disperse into the service environment (water in this case), and the need to separate them after use is recognized as one of the major drawbacks of deploying them in real water or wastewater systems. This has prompted some researchers to investigate options of supporting Pd nanocatalysts on larger membranes so as to enable insertion in and out of the water without the need for additional packaging or separation technologies to retrieve the materials from water. Membrane technologies have been widely applied into industry, as well as extensively studied at lab scale, especially in the field of wastewater treatment and environment protection. Membrane supports could enhance the recovery and durability of metal nanoparticles, as well as reduce the detrimental deactivation and aggregation of nanoparticles [129,130]. As discussed, Palladium possesses the capability to dissociate hydrogen as well as to cleave the carbon-halogen bond. Since a large number of emerging contaminants are halogenated, integrating palladium into membrane technology is a very promising approach for water treatment. [131,132,133]. A brief overview of the results to date on these types of materials is presented here.

#### 3.2.1. Polymeric Membranes 

In water purification technology, polymeric membranes have advantages of low cost, formability and scalability. Membranes are typically made of highly hydrophobic polymers in order to maintain structural integrity during the water treatment process. Since membrane porosities can be engineered to a very significant extent, molecular scale separation is possible for some pollutants through the physical structure of the membrane itself [134,135,136]. 

Two types of membranes have been created: Those with PdNPs attached to the surface and those where the PdNP is encapsulated inside the polymer matrix. There are advantages and disadvantages to both: Nanoparticles attached to the outer surface interact fully with the water, but also need to remain strongly attached and catalytically active throughout their service life. Hence, durability and reusability tests become very important. PdNPs that are encapsulated inside the matrix may be less prone to detachment, leaching and catalytic poisoning, but they may lose significant catalytic capabilities due to their surfaces not being directly exposed to the water being treated.

An example of membranes with PdNP attached to the outer surface was demonstrated by Smuleac et al. [137]. They deposited PdNPs on FeNPs, which were, in turn, immobilized on a polyacrylic acid, PAA-coated polyvinylidene fluoride (PVDF) membranes. This was accomplished via in-situ green synthesis using tea extract as a reducing and capping agent. Trichloroethylene (TCE) was used as a model pollutant compound to test these membranes, and successful destruction of the TCE was reported. The surface normalized rate constant was fond to be 0.005 L/m^2^h for Fe, which increased to 0.008 L/m^2^h for the Pd-enriched Fe. The catalyst activity was maintained even after three months of use.

**Figure 4:**
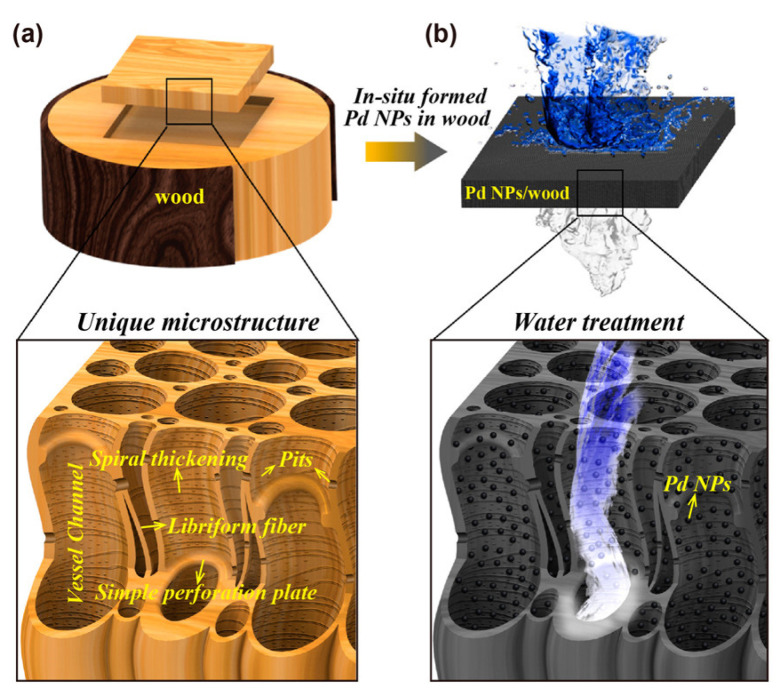
Basswood decorated with Pd NPs for water treatment. The zoom-in images show: (**a**) mesoporous structure of the wood perpendicular to the growth direction; (**b**) in-situ formed Pd NPs in wood where lignin acts as the reducing agent. “Reproduced with permission from [138]. American Chemical Society, 2017”.

Another example of externally attached PdNP can be seen in the study by Chen et al. who used mesoporous wood as a natural membrane and decorated that with PdNPs to achieve wastewater treatment. A schematic of this structure is shown in Figure 4. The abundant cellulose and lignin in the inner membrane of wood provides nucleation sites for a reduction in precursors and the formation of PdNPs. The authors used methylene blue as the model compound. A flowing test was performed, and the treatment rate was found to be 1 × 10^5^ L·m^−2^ h^−1^ with over 99.8% MB removal efficiency [138]. However, the durability and reusability of these materials have not been discussed in these publications, and are hence difficult to gauge. 

Kuvarega et al. shows an example of Pd-NPs encapsulated inside a polymer that enables interaction with water. They synthesized a nitrogen and palladium co-doped polysulfone(PSf)/TiO_2_ membrane The Pd was embedded inside the PSf/TiO_2_ membrane via a phase inversion method. TiO_2_ nanoparticles are photocatalytic and hydrophilic. They would enable water permeation into the encapsulated Pd NPs, resulting in catalytic interaction. They investigated photocatalytic degradation of dye (eosin yellow) using this material and significant dye removal (up to 97%) was achieved after 4 h of visible light irradiation [140].

In summary, several approaches of supporting palladium nanoparticles on polymeric membranes have been investigated for water treatment applications. However, additional studies are needed to gauge their practicality in real-world systems. Membrane fouling, caused by accumulations of contaminants on the surface or inside the pores, are important in polymer membranes. Their impact on nanocatalyst attachment and activities need to be understood, and additional surface modifications may be needed. Moreover, several polymer formulations have chemical incompatibilities with process solutions. This is especially true for wastewater or sludge that may contain high concentrations of multiple organic compounds. In such environments, the membrane materials may dissolve, swell, or weaken to the extent that their lifetimes become unacceptably short. Once the integrity of the whole material is compromised, either through membrane degradation or nanoparticle leaching, concerns of possible secondary pollution from membrane components become important.

To overcome these potential limitations, PdNP can be supported on continuous membranes of bio-inert and ecofriendly materials such as carbon. That has led our research team to focus on design, synthesis and fabrication of continuous carbon solids for water treatment, and an overview of relevant results are summarized in the next section.

#### 3.2.2. Continuous Carbon Structures

Advantages of carbon supports, in general, have been elaborated upon earlier in this paper. In the case of water purification, the additional advantage is that graphitic carbon serves as a very effective adsorbent for a wide variety of organic pollutants. In fact, adsorption by commercially available activated carbon is one of the most wide-spread approach of treating many organic contaminants in water such as volatile organic solvents [139]. At laboratory scales, it is also known that isolated nanostructures of carbon such as CNT and Graphene, as discussed in an earlier section, are very effective adsorbents. Many of the halogenated and unsaturated hydrocarbons contaminants could be degradable by palladium, and those compounds that include but are not limited to halogenated alkanes (e.g., Chlorofluorocarbons and hydrochlorofluorocarbons), ethylenes (e.g., TCE, tetrachloroethylene); and aromatics (e.g., Polychlorinated biphenyls); Polycyclic aromatic hydrocarbons (PAHs); formate/carbonate; and oxidized nitrogen species [141,142,143]. Therefore, incorporating Pd on carbon supports will combine the advantages of adsorbent and catalyst on the same membrane, hence enhancing process effectiveness. However, the major shortcoming of granulated carbon, CNT and different forms of Graphene is disintegration in the service environment, as pointed out in the earlier sections. This creates a need for follow-up processing such as separation, followed by recycling, reuse, or disposal of the used catalyst. 

Continuous solid carbon membranes with hierarchical bio-mimetic structures offer the advantages of carbon nanotubes (CNT) without the risks of disintegration and environmental dispersion. These consist of a porous carbon substrate enriched with a carpet-like layer of covalently bonded and vertically aligned CNT, which in turn support the ultra-fine palladium nanoparticles (PdNP). 

Different grades of porous carbon substrates materials are commercially available, making it possible for base substrates to be matched with the structural needs of the catalyst and water flow conditions. These include: (i) Rigid Foams—two types of rigid foams are common: cellular foams having spherical interconnected pores (in the range of 50 to 78% porosity), and reticulated foam consisting of interconnected struts, that can offer significantly higher porosities (in the range of 10 pores per inch (PPI) to 100 PPI, 97%).; (ii) woven carbon fiber fabric (CFC)—these are available with a wide variety of weaving patterns, porosities and permeability [144,145,146,147,148]. 

**Figure 5:**
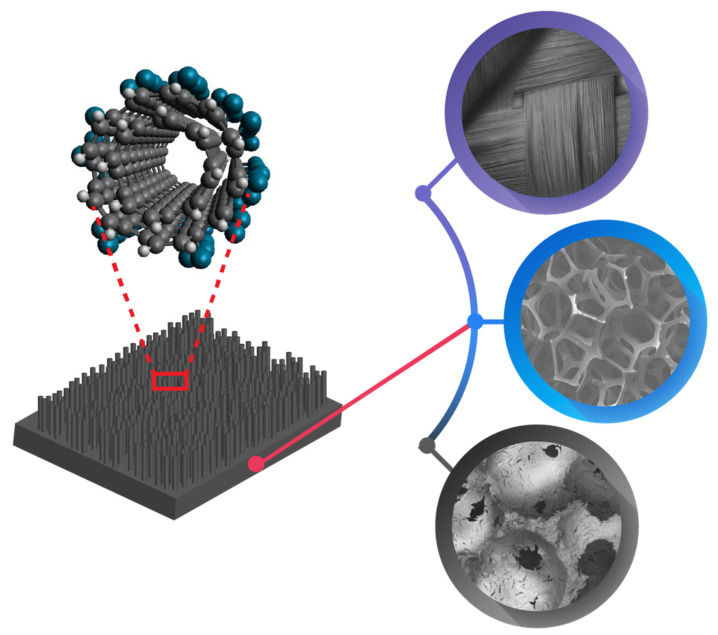
Schematic of hierarchical structure, where carpet-like arrays of CNT are strongly attached to a porous carbon substrate. Carbon substrate can be flexible carbon fiber cloth (**right-top**), reticulated vitreous carbon foam (**right-middle**) or micro cellular carbon foam (**right-bottom**).

**Figure 6:**
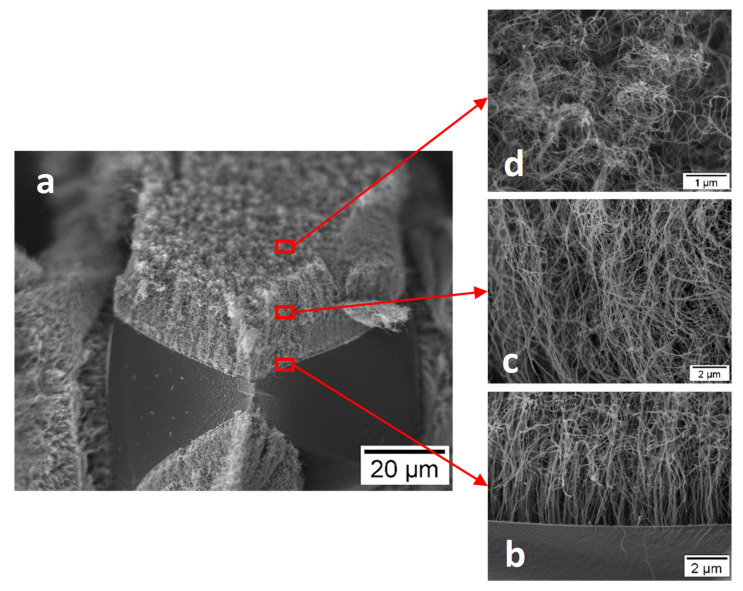

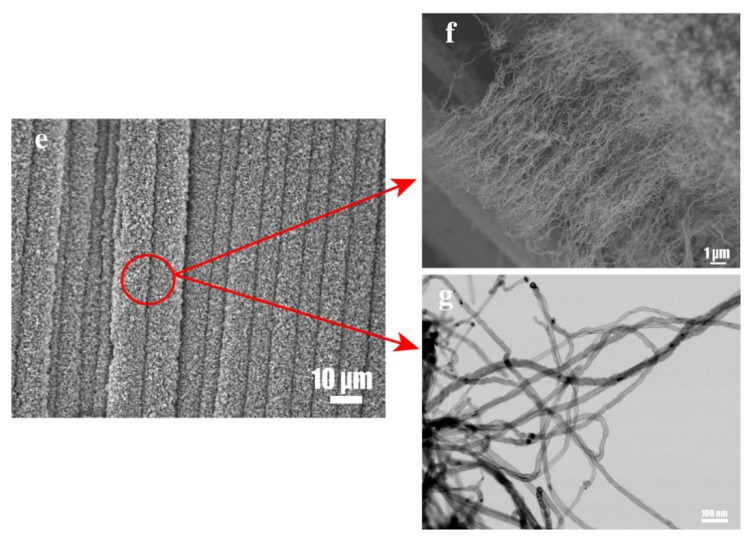
Supported PdNPs on CNT grafted carbon foam and carbon fabric. (**a**–**d**) aligned CNT carpet on RVC foam at different magnification, (**a**) low magnification overview, (**b**–**d**) CNT arrays entanglement level increase through the height of carpet, (**b**) root, (**c**) middle, (**d**) top [154]). (**e**–**g**) CNT carpet covered carbon fiber cloth. (**e**) low magnification micrograph of the CNT arrays. (**f**) aligned CNT arrys filling the spaces between the carbon fiber filament. (**g**) transmisison mode of CNT showing multiwall CNT features with uniform diameter distribution. “Reproduced with permission from [148]. Elsevier, 2021”.

CNT arrays are attached to the porous carbon foam or fabric using a two-step technique described in earlier papers [149,150,151]. The porous carbon substrate was first processed via plasma enhanced chemical vapor deposition (CVD) to create a silica buffer layer, which enabled nucleation and growth of CNT arrays through a floating catalyst chemical vapor deposition (CVD) process. It was shown that the CNT carpet was strongly attached onto the substrate through covalent C-Si bonds. Figure 5 shows a schematic of these structures and Figure 6 shows microstructures obtained from some of these materials. 

It was shown that attachment of the CNT carpet could easily increase the specific surface area (SSA) of these materials by several orders of magnitude, controllable by processing parameters and deposition times. Microstructural studies of CNT distribution and morphology was combined with physical measurements to make geometrical estimates of SSA, which matched very well with gas adsorption studies using Brunauer–Emmett–Teller (BET) analyses [152]. It was also seen that the entire CNT surface was available for adsorption of molecular species from the surrounding environment, as tested for by Methylene Blue adsorption in water. [153].

**Figure 7:**
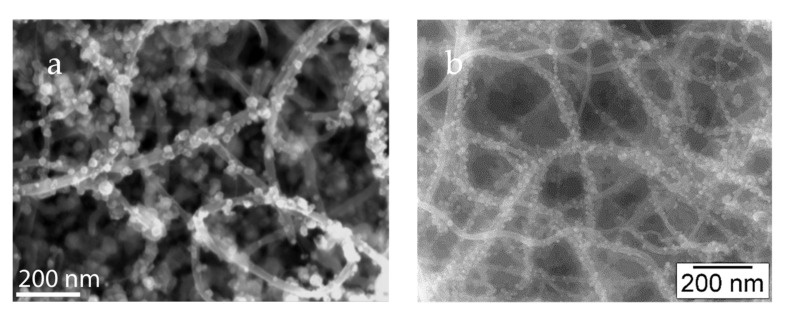
(**a**,**b**) SEM microstructure of PdNPs well attached on the CNT with fine distribution “Reproduced with permission from [155]. Elsevier, 2012”.

The hierarchical substrates (carbon foam or fabric enriched with carpet-like arrays of CNT) were used for the attachment of palladium nanoparticles. This was achieved through dip coating in precursor solution containing diluted tetra-amine palladium (II) nitrate solution, followed by heat treatment at 400 °C in a reducing environment using hydrogen as the reducing gas [155]. Figure 7 shows the micrograph of deposited PdNPs on CNTs carpets attached to graphitic foam. Particles are well distributed throughout the CNT surface and particle agglomeration is avoided. 

Robustness and reusability of these materials were also tested to ensure the mechanical integrality of the synthesized catalyst. It was seen that when this material is subjected to extreme mechanical stresses leading to complete substrate failure, the CNT layer still remains attached to the top graphitic substrate with the metallic nanoparticles intact [147,149], indicating that the catalytic film will not be destroyed during operation. Subsequent studies routinely showed that hours and weeks of vigorous rotation (32 rpm) did not create any detectable change in structure and Pd content. Model volatile organic halogenated compounds that have been degraded using these materials include Carbon tetrachloride (CTC), trichloroethane (TCA), trichloroethylene (TCE). This was accomplished by catalytic cleavage of carbon–halogen bonds enabled by Pd nanoparticles in the presence of hydrogen. A balanced 5% H_2_ and N_2_ gas mixture was bubbled through the reactor as a hydrogen source. Complete degradation of each of these pollutants was accomplished by this material at room temperature [154]. 

**Figure 8:**
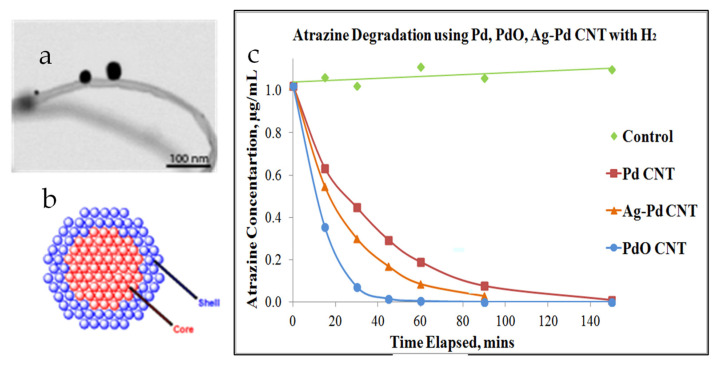
Palladium nanoparticles attached to CNT carpets: (**a**) scanning transmission electron microscopy (STEM) image showing microstructure of Pd nanoparticles attached on CNT; (**b**) Atomistic model of core-shell type architecture of palladium based nanocatalyst; (**c**) Comparative Atrazine degradation profile with different nanocatalyst “Reproduced with permission from [156]. Elsevier, 2012”.

Additional refinement of the Pd-nanoparticles was made to understand if and how such materials can be tailored in the future. The Pd nanocatalysts attached to the CNT carpets were further treated to form core-shell type architectures. Three types of palladium-based nanocatalysts were created: metallic zero-valent Pd (as deposited); Pd core particles with silver shell; and PdO shell on the metallic Pd core. These were tested for degradation of a chlorinated herbicide (atrazine). Figure 8 summarizes some of the results. Surface chemical states of the outer layers were obtained by X-ray photoelectron spectroscopy (XPS), and comparisons of spectra between metallic and oxide coated palladium are performed. XPS data was supplemented with Energy Dispersive Spectroscopy using X-Rays (EDAX), where analysis depth is greater than particle size, providing average composition over the entire particle size. Combining these with scanning electron microscopy (SEM)/transmission electron microscope (TEM) images for overall nanoparticle morphology and X-ray diffraction (XRD) data for predominant crystalline facets, it was concluded that these nanoparticles have a metallic core of predominantly (111) palladium facets and two of them have shells of PdO and silver (Ag), respectively. The plot on the right of Figure 8 indicates that the three variations in Pd nanocatalyst result in distinctly different catalytic degradation rates for Atrazine in water. Daughter products analyzed using gas chromatography-mass spectroscopy (GCMS) and high performance liquid chromatography (HPLC) indicate two possible reaction pathways, each leading to less toxic by-products. The result suggested PdO (Pd^2+^) NPs had the highest catalytic activity, with Pd-Ag bimetallic in second, followed by metallic PdNPs in the dechlorination process [156]. This opens the possibility of incorporating advanced Pd-based catalysts, such as bimetallic and core-shell type particles. In the case of the Pd-Ag bimetallic catalyst, it is feasible to target both organic pollutants and bacteria simultaneously. 

**Figure 9:**
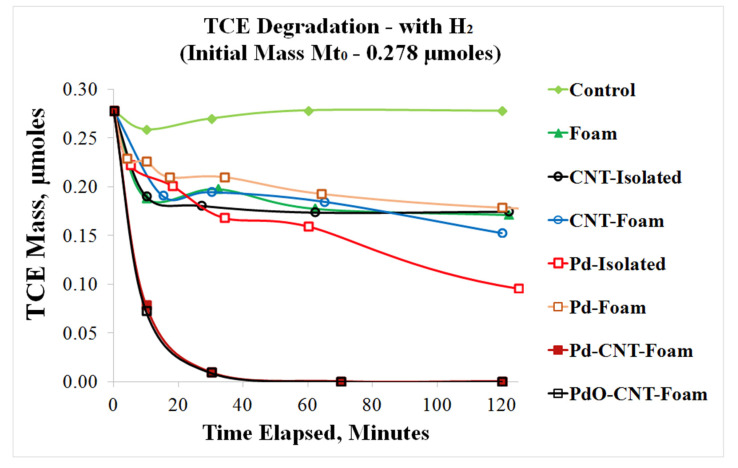
Dechlorination profile of TCE using various catalysts. It must be noted that the nanoparticles of Pd and PdO attached to CNT-Foam show rapid and complete degradation that is not seen in any of the controls, including isolated Pd (loose nanoparticles) “Reproduced with permission from [157]. MDPI, 2016”.

These materials were tested for degradation of trichloroethylene (TCE), two concentrations of TCE, one part per million (ppm) and five ppm, were tested and TCE degradation was complete in both cases, the final product being n-butane. In fact, this material appears to be the only catalyst structure reported in the literature that accomplishes complete degradation of TCE in such a short time [154]. Figure 9 shows the results for one ppm (0.278 μmol) TCE. Here, the catalytic rate of this hierarchical hybrid material was compared with those of loose Pd nanoparticles (Pd-Isolated) as well as isolated CNT and bare CNT-Foam adsorbents. It was seen that the hierarchical material (Pd-CNT-Foam) was significantly more effective than all others, including isolated Pd nanoparticles. While improvement over other materials are not surprising, the fact that this architecture is performing better than isolated nanoparticles is noteworthy, since isolated nanocatalysts dispersed in the fluid medium should, in principle, provide the highest level of interaction, even if they are not practical for real-life applications. While the TCE concentration was brought up to five ppm, authors observed the catalytic activity of PdO-CNT-Foam sample surpassed the Pd-CNT-Foam counterpart. Repeatability tests were performed up to three cycles, and results indicated the catalytic activity was maintained. The observed improvement caused by the hierarchical hybrid architecture may be attributed to the fact that this structure also prevents nanoparticle agglomeration with time, which is a high possibility for isolated nanoparticles [154,157]. 

**Figure 10:**
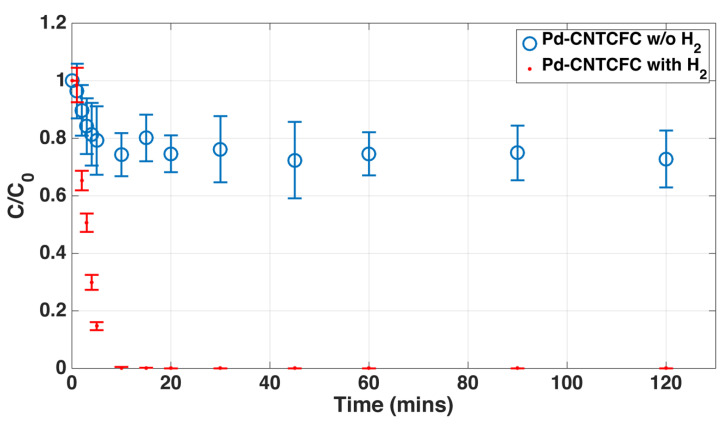
TCS catalytic degradation in aqueous environment, nitrogen balanced hydrogen (5% H_2_) mixture was supplied into the reactor, and the pressure was kept at a constant of 1 atm. “Reproduced with permission from [158]. Elsevier, 2021”.

Wang et al. [158] extended the technique to grow Pd nanoparticles on CNT carpet attached to flexible carbon fabric substrates. The idea was to test if hierarchical hybrid Pd nanocatalysts can be dispersed as flexible sheets, if needed for special applications. Triclosan (TCS) a chlorinated aromatic compound that has functional groups representing both ethers and phenols, was chosen as a model pollutant. The added complication of this contaminant is that it has an aromatic component, which is harder to target compared to purely aliphatic chains. The enthalpy of formation for TCS is at −209 kJ/mol, while TCS is around −20 kJ/mol (−18 ± 2), and therefore, those chlorine atoms on the TCS are more difficult to remove. The saturation solubility of TCS in water is only 10 ppm, and these materials degraded the contaminant at that level almost instantly; a complete degradation in less than 10 min, with just 1 wt% of the PdNPs loading, as shown in Figure 10. Repeatability test in the aqueous solution showed the removal capacity was maintained fully for up to four cycles. To fully evaluate TCS kinetic degradation rates in more detail, up to 50 ppm TCS solution was prepared in methanol. The hybrid catalyst membrane showed a rate constant of 87 mmol/min/g_Pd_ in a methanol environment, which is orders of magnitude higher than the previous work carried out by other groups using PdNPs as a catalyst to degrade TCS [125,159]. The degradation byproducts analyzed over time indicated that the C-Cl bonds were cleaved from the parent TCS molecule one at a time, showing intermediates containing two and one Cl per molecule, respectively, in the intermediates. Eventually, all the intermediates products disappeared, leaving only the final hydrocarbon compound (2-Phenoxyphenol) with no Chlorine. 

## 4. Concluding Remarks

An overview of recent advances in matrix-supported pallidum nanoparticles has been provided. These hybrid materials have the potential to impact various industries including pharmaceutical, biomedical, oil and gas, food and beverage, energy, and environment. For water treatment applications, Pd nanoparticles can provide many advantages, including their capability to cleave the persistent carbon-halogen bonds seen in many emerging pollutants. In this paper, we have provided an overview of three different types of packaging options for nano-palladium materials suitable for the degradation of emerging contaminants in water. 

Novel organic supports (e.g., polymer cages, dendrimers and metal-organic frameworks) offer some unique advantages in designing the next generation palladium nanocatalysts suitable for degrading current and emerging water contaminants. Several inorganic compounds such as porous oxides like zeolites and silica, photocatalysts, as well as magnetic particles can also provide functional advantages ranging from heat and chemical stability to bandgap modulation, photocatalytic functions, and magnetic separation. Carbonaceous supports such as granular carbon with different levels of activation, nanographene particles and carbon nanotubes provide a wide range of stability in both acid and base environments, along with the unique catalyst-support interactions offered by 2D graphitic planes. 

While nanomaterials and specialty particulate products offer several advantages, it should be noted that disintegration and leaching into the service environment, as well as possibility of agglomeration have limited their wider practical applications. This will require additional steps for material retrieval after use and can raise reusability issues. These considerations have led the research community to investigate palladium nanoparticles supported on larger membranes (e.g., polymeric membranes). 

These considerations have led to studies where palladium nanoparticles are attached to larger continuous solid membranes. Polymeric membranes are ubiquitous in water filtration applications. They offer the advantages of easy fabrication, low capital cost, high selectivity and permeability, and hence are good candidates for supporting nanoparticles, in this case, the PdNPs. However, polymer membranes also have few drawbacks: their long term stability in harsh environments (e.g., high temperature and extreme pH) is questionable, and their chemical resistance may be inadequate, with risks to structural integrity, in certain solvents. 

To overcome current membrane limitations (e.g., stability in a harsh environment and fouling), an inert and ecofriendly material such as a continuous carbon membrane should be considered. By creating biomimetic multiscale carbon structures as a support (CNT attached porous carbon) for a nanocatalyst, the resultant hybrid material can have good resistance to chemical/biological degradation, high temperature stability and enhanced catalytic activities. Additionally, the continuous carbon membrane can have tailor-designed structural properties with tunable water permeability and wettability.

## Data Availability

Data sharing not applicable.
